# Complete mitochondrial genome of near threatened fish species *Osteobrama belangeri* (Cypriniformes: Cyprinidae)

**DOI:** 10.1080/23802359.2017.1331327

**Published:** 2017-05-27

**Authors:** Anindya Sundar Barman, Mamta Singh, Pramod Kumar Pandey

**Affiliations:** College of Fisheries, Central Agricultural University, Tripura, India

**Keywords:** *O. belangeri*, mitochondrial genome, phylogenetics

## Abstract

The complete mitochondrial genome of *Osteobrama belangeri* was obtained, using Illumina high-throughput NextSeq 500 with 2 × 150 bp sequencing of mitochondrial DNA. The mitochondrial genome of *O. belangeri* was 16,602 bp in length (GenBank Accession No. KY887473), comprised of 13 protein coding genes, 22 tRNA genes, 2 rRNA genes and a control region, i.e. D-loop. In present mitogenome, 11 short sequence repeats were identified and validated *in silico.* The arrangement of genes was found identical to other Cypriniformes fish mitogenomes, available in NCBI database. Phylogenetic relationship established in the present study also supported that genus *Osteobrama* is member of subfamily Cyprininae (tribe: smiliogastrini) not Cultrinae, which provide useful insights into taxonomic status of the genus.

*Osteobrama belangeri* (order: Cypriniformes, family: Cyprinidae), a medium size carp, is locally known as ‘Pengba’ in the Indian state of Manipur. Owing to the taste (Manipur delicacy), religious importance during local festival (Ningol Chakouba) and near threatened IUCN status (Vishwanath 2010), the fish was declared as state fish of Manipur in the year 2007. This species is endemic to Manipur state of India, Myanmar and Yunnan Province of China. In past years, this fish formed a big fishery resource of Loktak Lake (largest freshwater Lake of North-East India). But due to habitat loss and breeding ground destruction, its population declined to the drastic level in Loktak Lake and central plains of Manipur.

For the present study, samples of *O. belangeri* were obtained from culture pond of College of Fisheries, Tripura, India (23°54.248’N, 91°18.465’E) in the month of November 2016 and maintained at the Fish Museum (specimen voucher no. OB-WM-MN01) of College of Fisheries (Central Agricultural University), Tripura, India. Total mitochondrial DNA was isolated from liver tissue and sequenced, using illumine high-throughput NextSeq 500 (Illumina, Inc., CA) with 2 × 150 bp paired end chemistry. A total of 9,287,508 reads were obtained and mapped to the 2141 fish mitochondrial genomes downloaded from MitoFish. Mapped reads were *de novo* assembled into scaffolds, using Velvet version 1.2.10 (Zerbino & Birney 2008) and gene prediction and annotation of the mitogenome were done with the help of MitoAnnotator (and short sequence repeats (SSR) were identified, using MISA version 1.0 (Thiel et al. 2003). Maximum likelihood (ML) phylogenetic tree was (Iwasaki et al. 2013) constructed with the help of MEGA version 7.0 (Kumar et al. 2016).

The complete mitochondrial genome of *O. belangeri* is 16602 bp in length (GenBank Accession No. KY887473), comprising 13 protein coding genes, 22 tRNA genes, 2 rRNA genes and a 930-bp-long control region, i.e. D-loop. Majority of genes were found on H strand, except ND6, tRNA^Glu^, tRNA^Pr^°, tRNA^Gln^, tRNA^Ala^, tRNA^Asn^, tRNA^Cys^, tRNA^Tyr^ and tRNA^Ser^ which were encoded on L strand. GC % of protein coding, tRNA, rRNA genes and D-loop region was found to be 38.72, 42.42, 42.90 and 32.10, respectively. A total of 11 SSR were identified and validated *in silico*.

The phylogenetic relationship of *O. belangeri* was established with 19 closely related cyprinid species and 2 outgroup species, using ML method based on the Kimura 2-parameter model (Kimura 1980). The tree with the highest log likelihood (−119622.40) is shown in Figure 1. Initial trees for the heuristic search were obtained automatically by applying neighbour-joining and BioNJ algorithms to a matrix of pairwise distances estimated, using the maximum composite likelihood (MCL) approach and then selecting the topology with superior log likelihood value. Initially, *O. belangeri* was considered as member of family Cyprinidae and subfamily Cultrinae (Howes 1991; Arai 2011). But a recent study based on mitochondrial genes, whole mitochondrial genome and nuclear gene RAG1, placed this species under subfamily Cyprininae and tribe smiliogastrini (Yang et al. 2015). Phylogenetic relationship established in the present study also found concordant with previous report of Yang et al. (2015). The ML tree showed clustering of *O. belangeri* with genus *Systomus*, *Barbodes* and *Barbus* which are the member of tribe smiliogastrini of subfamily Cyprininae with 100% bootstrap support value (Figure 1).

**Figure 1.**
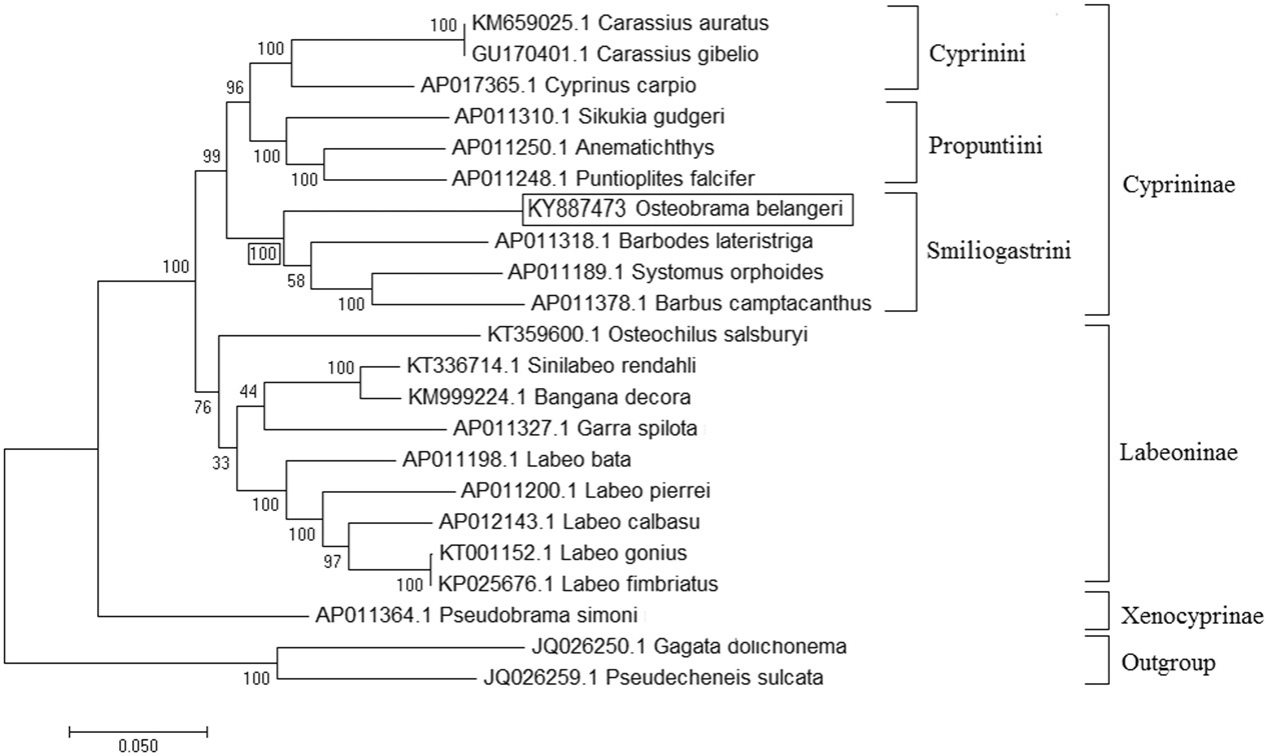
Maximum likelihood phylogenetic tree of closely related 20 cyprinids and two outgroup species. The percentage of trees in which the associated taxa clustered together are shown next to the branches and tree is drawn to scale, with branch lengths measured in the number of substitutions per site.
